# Indwelling pleural catheter and successful autopleurodesis of refractory inflammatory lupus effusion

**DOI:** 10.1002/rcr2.621

**Published:** 2020-07-15

**Authors:** Boon Hau Ng, Nik Nuratiqah Nik Abeed, Mohamed Faisal Abdul Hamid, Chun Ian Soo, Hsueh Jing Low, Andrea Yu‐Lin Ban

**Affiliations:** ^1^ Pulmonology Unit, Department of Medicine, Faculty of Medicine Universiti Kebangsaan Malaysia Medical Centre Kuala Lumpur Malaysia; ^2^ Department of Anaesthesiology and Critical Care, Faculty of Medicine Universiti Kebangsaan Malaysia Medical Centre Kuala Lumpur Malaysia

**Keywords:** Indwelling pleural catheter, lupus effusion, spontaneous pleurodesis, systemic lupus erythematosus

## Abstract

Indwelling pleural catheter (IPC) is a useful tool for refractory malignant pleural effusions (MPEs). It allows palliation by intermittent symptomatic relief of the effusion and improves quality of life. Its use in benign pleural effusions comes mainly from retrospective studies, case series, and case reports. Lupus effusion is common, causes minimal symptoms, and usually responds to either steroid therapy or immunosuppressants. Refractory lupus effusion is less common and treatment may require invasive surgical pleurectomy. We describe a 52‐year‐old woman whose first presentation of systemic lupus erythematosus (SLE) was a pleural effusion refractory to steroids and immunosuppressants. She successfully achieved spontaneous pleurodesis with intermittent IPC drainage at three months.

## Graphical abstract

Indwelling pleural catheter (IPC) is a useful tool for refractory malignant pleural effusions (MPEs). Its use in benign pleural effusions comes mainly from retrospective studies, case series, and case reports. Lupus effusion is common, causes minimal symptoms, and usually responds to either steroid therapy or immunosuppressants. We describe a 52‐year‐old woman whose first presentation of systemic lupus erythematosus (SLE) was a pleural effusion refractory to steroids and immunosuppressants. She successfully achieved spontaneous pleurodesis with intermittent IPC drainage at three months.

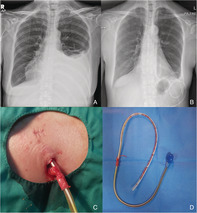

## Introduction

Indwelling pleural catheter (IPC) is an effective treatment for recurrent malignant pleural effusions (MPEs). It is useful for relief of symptoms caused by rapid accumulation of fluid that would otherwise require repeated pleurocentesis. It is a one‐day outpatient procedure and drainage can be done at home by a trained family member. In principle, IPC can also be beneficial in refractory non‐MPEs (NMPEs). Data on IPC usage in refractory NMPE come mainly from retrospective studies and case series. We report our experience of IPC in refractory lupus effusion.

## Case Report

**Figure 1:**
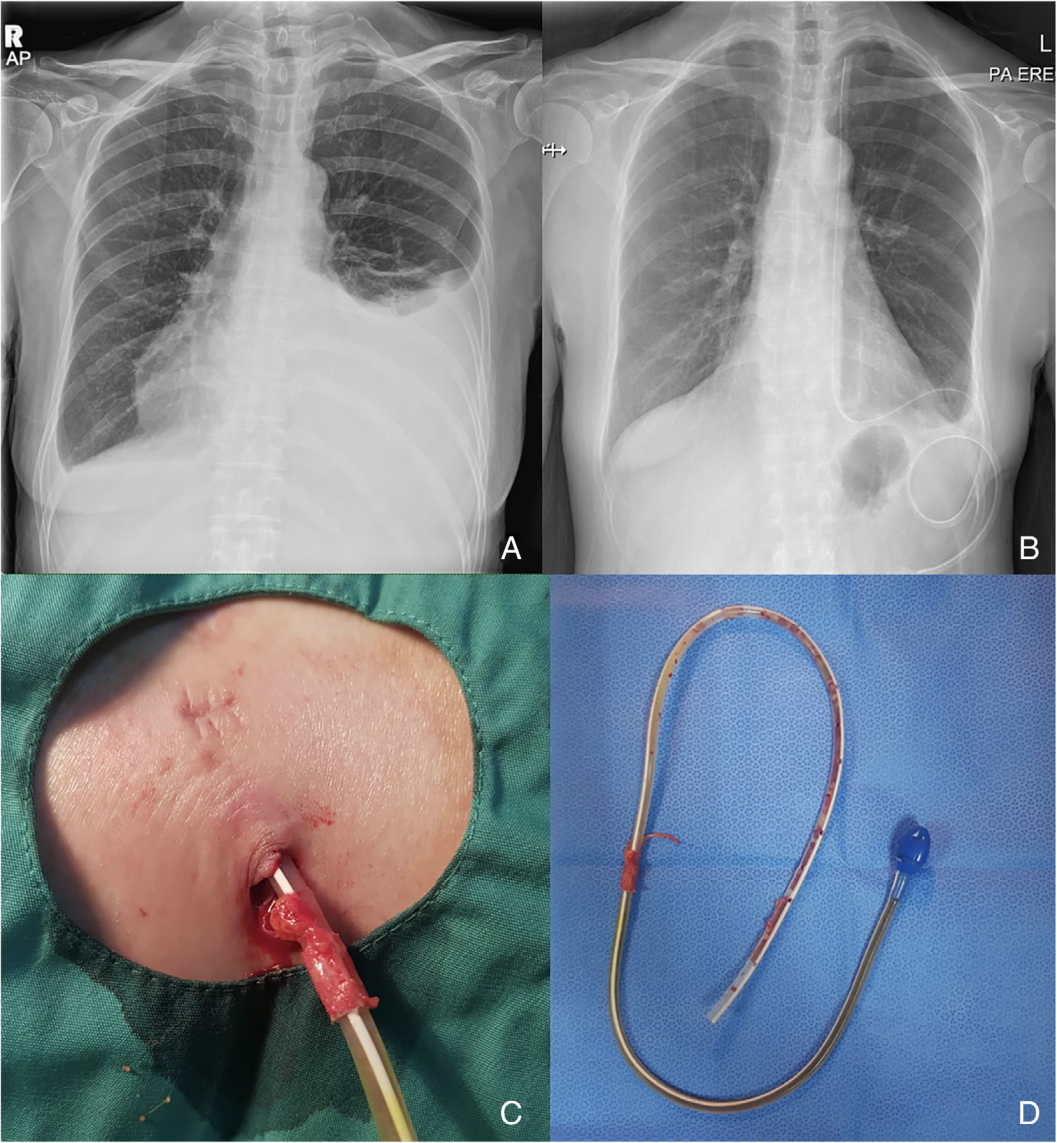
(A) Chest radiograph on admission showed moderate left pleural effusion. (B) Resolution of left pleural effusion with IPC insertion in situ at three months. (C) Removal of indwelling pleural catheter (IPC) with fibrous tissue engulfing the cuff. (D) Post‐removal intact IPC.

A 52‐year‐old woman presented with a two‐week history of progressive dyspnoea. She had a history of medical pleuroscopy two weeks earlier at a different centre. This was reported as mild inflamed pleura. Clinically, she was mildly tachypnoeic with oxygen saturation of 92% on room air. Chest radiograph showed a moderate pleural effusion (Fig. 1A). Pleurocentesis was performed and 1500 mL of fluid was drained. Pleural fluid analysis showed an exudative effusion with both pleural fluid/serum protein and pleural fluid/serum lactate dehydrogenase (LDH) ratio of 0.66. The pleural fluid protein was 37 mg/L, LDH was 133 IU/L, and glucose was 6.4 g/L. Cytology was negative for malignant cells and Gram staining and culture results were negative. Urine protein creatinine index was 0.17 g/mmol creatinine with normal serum creatinine. Echocardiogram was also normal.

A repeat pleuroscopy showed a uniformly inflamed pleura. Histopathological examination of the pleural biopsy demonstrated chronic inflammatory infiltrates composed of mainly lymphocytes, plasma cells, and histiocytes. Pleural fluid adenosine deaminase (ADA) was 2.8 U/L, and the Xpert MTB/RIF Ultra (Cepheid, USA) was not detected. We did not send the pleural fluid analysis for anti‐nuclear antibodies (ANA). Serum ANA was positive with a titre of 1:640 and anti‐double‐stranded DNA was elevated at 81.10 IU/mL (positive is >75) with reduced complement levels (C3: 35 mg/dL and C4: 3.9 mg/dL). On the basis of these results, we made a diagnosis of systemic lupus erythematosus (SLE) with lupus pleurisy and lupus nephritis.

**Figure 2:**
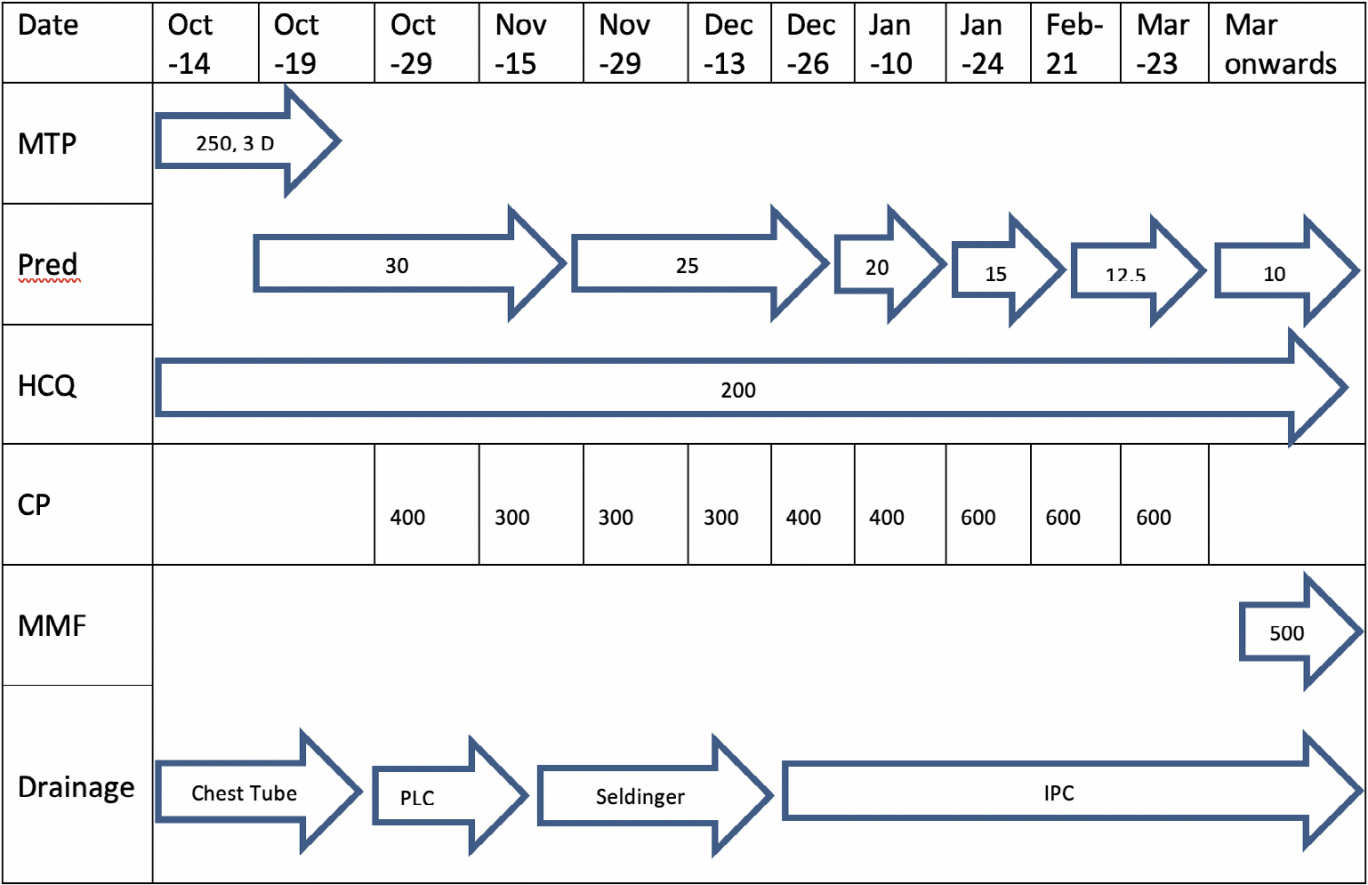
Drug history and frequency of recurrence of pleural effusion. CP, cyclophosphamide; D, day; HCQ, hydroxychloroquine; MMF, mycophenolate mofetil; MTP, methylprednisolone; PLC, pleurocentesis, Pred, prednisolone (note: the dosage is in mg and the frequency is daily except MMF which was given twice a day).

She was treated with three days of intravenous (i.v.) methylprednisolone (MTP) 250 mg daily, followed by oral prednisolone of 1 mg/kg daily and i.v. cyclophosphamide (CP) (Fig. 2). She required one therapeutic pleurocentesis which drained 1500 mL. One month later, the patient developed a symptomatic recurrence of the pleural effusion and required Seldinger chest drain. The drainage continued despite cumulative CP dose of 1.7 g and maintenance oral prednisolone. The patient did not want any surgical intervention. We chose IPC over talc slurry pleurodesis due to the high output chest drainage.

She performed regular IPC drainage of 300–400 mL per session, three times per week at home with no complications of site infection nor blockage of the IPC. She achieved spontaneous pleurodesis (Fig. 1B) at three months post insertion and the IPC was successfully removed with no complications (Fig. 1C, D).

## Discussion

Pleural effusion in SLE is common. Pleuritis is the main cause but other disease‐related problems such as nephrotic syndrome, congestive cardiac failure, and pulmonary embolism can also be the cause. Lupus effusion can be the first manifestation of the disease as in our case or it can be associated with other organ involvement. It is usually mild to moderate with only a few reported cases of massive effusions [1].

The effusion is usually mild, exudative, and lymphocytic with the presence of ANA and anti‐DNA antibodies. Treatment is individualised according to the degree of symptoms. Small, asymptomatic effusions do not need pleurocentesis and usually responds to non‐steroidal anti‐inflammatory drugs and steroid therapy. Azathioprine and hydroxychloroquine may be added. The majority of patients will respond to therapy within days [1]. Refractory lupus effusion is rare. The reported treatment modalities include systemic and local therapy such as tetracycline or talc pleurodesis and even a surgical approach like pleurectomy.

IPC is a multi‐fenestrated small‐bore silicone chest drain with a fibrotic cuff and one‐way valve. IPC usage in NMPE is limited to retrospective cohort studies, case series, and case reports. IPC is not widely used in NMPE and the low numbers make it difficult to report on its efficacy [2]. Patients with NMPE appear to have longer IPC duration (110 vs. 36 days) compared to MPE [3]. This may be due to longer survival duration in patients with NMPE. Patient symptom improvement while on IPC appears to be comparable to that seen in MPE.

The success rate of spontaneous pleurodesis following IPC insertion ranges from 33% to 60% [4]. The average time to pleurodesis appears to be longer in NMPE compared to MPE (95 vs. 36 days) [3]. The goal of IPC is control of dyspnoea without the need for recurrent pleurocentesis. Absolute contraindications of IPC include an inability for the patient or their family for IPC care, ongoing pleural infection, uncorrected coagulopathy, or cutaneous chest wall infection.

The common complications are related to malfunctions of the IPC and infections. Pleural infection rates are low (2.8%) and usually occurs two months after insertion [5]. Pleural infection after IPC is managed conservatively with continuous drainage and i.v. antibiotic. Drainage removal is rarely needed. Blockage can be managed by either reposition of the IPC or instillation of the intrapleural fibrinolytic agents.

The conventional methods for managing recurrent or refractory pleural effusions are usually chest tube insertion (with or without chemical pleurodesis) or repeating pleurocentesis. Surgical intervention, such as parietal pleurectomy or pleuroperitoneal shunting, may be considered in a suitable patient. IPC is an important alternative as it improves symptoms with no need for repeated pleurocentesis and has a manageable safety profile. It should be considered as a therapeutic option for refractory NMPE.

### Disclosure Statement

Appropriate written informed consent was obtained for publication of this case report and accompanying images.
